# Loneliness, Social Isolation and All-Cause Mortality in Older Adults

**DOI:** 10.1093/geroni/igab046.3326

**Published:** 2021-12-17

**Authors:** Timothy Barnes, Rifky Tkatch, Manik Ahuja, Laurie Albright, James Schaeffer, Charlotte Yeh

**Affiliations:** 1 UnitedHealth Group, Minnetonka, Minnesota, United States; 2 UnitedHealth Group, Oak Park, Michigan, United States; 3 UnitedHealth Group, Minneapolis, Minnesota, United States; 4 AARP Services, Inc., Washington, District of Columbia, United States

## Abstract

As distinct constructs, loneliness and social isolation have both been associated with mortality in older adults. Many studies have examined each construct separately; however, few have examined their impact together, especially within the U.S. Using data from a large sample of U.S. adults age 65+ (N=7,982), the effect of loneliness and social isolation on all-cause mortality was examined considering their separate and joint effects. Measures were based on the UCLA-3 Loneliness Scale and the Social Network Index (SNI). Loneliness was categorized as: Severe, moderate, or no loneliness. Social isolation (defined by the SNI) was categorized as: Limited, medium, or diverse social networks (SN). Cox proportional hazards regression models were performed. Among participants, there were 328 deaths after data collection (4.1%). In separate, adjusted models, loneliness (severe, HR=1.86, 95% CI: 1.43-2.41 and moderate, HR=1.51, 95% CI: 1.16-1.98) and social isolation (limited SN, HR=2.37, 95% CI: 1.72-3.27 and moderate SN, HR=1.55, 95% CI: 1.12-2.14) were both associated with mortality. Modeled together, loneliness (severe, HR=1.55, 95% CI: 1.18-2.04 and moderate, HR=1.40, 95% CI: 1.07-1.83) and social isolation (limited SN, HR=2.08, 95% CI: 1.49-2.89 and moderate SN, HR=1.46, 95% CI: 1.05-2.02) both remained significantly associated with all-cause mortality with limited SN as the stronger indicator. Results demonstrate that both loneliness and social isolation contribute to greater risk of mortality among older adults. Furthermore, individuals with limited SN are at greatest risk. As the COVID-19 pandemic continues, loneliness and social isolation should be targeted safely in efforts to reduce mortality risk among older adults.

